# Uptake and correlates of HIV testing among men in Malawi: evidence from a national population–based household survey

**DOI:** 10.1186/s12913-019-4031-3

**Published:** 2019-03-29

**Authors:** Chrispin Mandiwa, Bernadetta Namondwe

**Affiliations:** 1 Ministry of Health, South–West Zone Quality Management Office, P.O. Box 3, Blantyre, Malawi; 2 Kamuzu College of Nursing, University of Malawi, Lilongwe, Malawi; 3 Management Sciences for Health (MSH), Lilongwe, Malawi

**Keywords:** HIV testing, Men, Malawi

## Abstract

**Background:**

HIV testing is one of the key strategies in the HIV/AIDS prevention and control programmes. However, studies examining utilization of this service by men in Malawi are limited. The aim of this study was to assess the uptake and determinants of HIV testing among men in Malawi.

**Methods:**

Secondary data analysis was conducted on cross–sectional household data for 7478 men aged 15 to 54 years drawn from the 2015–16 Malawi Demographic and Health Survey. Descriptive statistics, bivariate and multivariable logistic regression analyses were performed to identify the socio–demographic, behavioral and health service related factors that are associated with HIV testing service utilisation by men in Malawi. All analyses were performed using the complex sample analysis procedure of the Statistical Package for the Social Sciences version 22.0 to account for the multistage sampling used in Demographic Health Survey.

**Results:**

Overall, 69.9% of the participants had ever been tested for HIV. The results indicate that age, region of residence, marital status, covered by health insurance, education and age at first sexual debut are significant predictors of HIV testing among men in Malawi. In particular, men who were in the age group 30–39 years (AOR = 3.00; 95% CI = 2.35–3.82), married (AOR = 3.03; 95% CI = 2.51–3.65), those with secondary or above education (AOR = 3.02; 95% CI = 2.33–3.91), and those who had health insurance (AOR = 1.66; 95% CI = 1.05–2.63) were likely to utilise HIV testing service than their counterparts.

**Conclusion:**

The findings suggest that HIV testing services and programmes need to target younger unmarried men aged 15–19, men with low level or no education and expand HIV testing services to the central and southern regions of Malawi. Targeting the undiagnosed men living with HIV in a timely manner is a crucial and necessary step not only for achieving the UNAIDS’ 90–90–90 targets but for individuals to benefit from antiretroviral treatment and to sustainably reduce population–level HIV transmission.

## Background

Malawi is making progress in the fight against HIV: The HIV prevalence for adult population aged 15–49 has declined over the past 5 years, from 10.6% in 2010 to 8.8% in 2016 [1]. New HIV infections have also dramatically declined from 98,000 new infections in 2005 to 36,000 in 2016 [2]. However, these estimates are still unacceptably high, and Malawi needs to make significant improvements to achieve the Joint United Nations Programme on HIV/AIDS (UNAIDS) 90–90-90 targets by 2020, which include 90% of people with HIV knowing their status, 90% of all people diagnosed with HIV infection receive sustained antiretroviral therapy (ART) and 90% of those on treatment being virally suppressed [3]. In order to reach the UNAIDS 90–90–90 targets it is critical that HIV testing services (HTS) be strategically expanded to diagnose many people living with HIV.

HIV testing is an essential strategy for reducing HIV related morbidity, mortality and may improve patient outcomes [4–6]. The World Health Organisation (WHO) and Centre for Disease Control (CDC) recognise HIV testing as a critical gateway to prevention of HIV transmission, treatment, care and other support services [7, 8] . HIV testing empowers individuals and couples to adopt measures to avoid the transmission or acquisition of HIV infection [9]. Besides, HIV testing provides access to HIV prevention services, including prevention of mother-to-child transmission (PMTCT) and male circumcision [10]. Furthermore, knowledge of HIV status is a necessary step for initiation of ART and also serves as the basis for accessing care as well as emotional support that enable individuals to cope with HIV–related anxiety and increasing motivation to avoid risky behaviours [11, 12].

Malawi has been implementing rapid HIV testing services since early 2000. The services are provided free of charge in public health facilities and some private clinics/hospitals [13]. Despite the availability of free HIV testing services, there has been low uptake of HIV testing services among men in Malawi. A recent Malawi population–based HIV impact assessment (MPHIA) estimates that 35% of men in Malawi have never tested for HIV [14]. Men are a key population disproportionately affected by HIV and represent an important group to engage in HIV testing and prevention services. Besides, men in Malawi are regarded as key decision–makers in the families and might influence the control of economic resources that are significant for HIV prevention and care. Thus, a better understanding of the factors influencing the uptake of HIV testing in this population is required to inform the development of strategies to scale up HIV testing among men in Malawi, and ultimately, prevent HIV infection and promote timely linkage to HIV treatment and care. For instance, this might highlight the specific categories of men who need to be targeted with more efforts in order to improve HIV testing uptake. Elsewhere, studies have found a positive association between the likelihood of men having tested for HIV and older age, marital status, higher income as well as higher educational [15–18]. To date, there have been no studies that have investigated the predictors of HIV testing among men in Malawi. Therefore, the aim of this study was to investigate factors associated with lifetime HIV testing among men in Malawi using a nationally representative sample.

## Methods

### Study design, data sources and population

Secondary data analysis was conducted on cross–sectional household data drawn from the 2015–16 Malawi Demographic and Health Survey (MDHS) which is the fifth national sample survey that aims to provide current estimates of basic demographic and health indicators at national, regional and district level. The survey was implemented by the National Statistical Office (NSO) in joint collaboration with the Ministry of Health. The International Classification of Functioning, Disability, and Health (ICF) provided technical support for the survey and stakeholders including the government of Malawi, the United States Agency for International Development (USAID), the United Nations Children’s Fund (UNICEF), and others provided financial support. The field work for data collection was carried out between October 2015 and February 2016. Samples were obtained using a multistage stratified sampling strategy in which 24,562 women of reproductive aged 15–49 years and 7478 men aged 15–54 years were interviewed separately. For the purpose of this study, our analyses included data for 7478 men which were collected using the men’s questionnaire. The questionnaire collected different types of information from the respondents on socio–demographic characteristics, sexual behaviour history, HIV/AIDS–related knowledge, and HIV/AIDS–related stigma. The methodology used in the 2015–2016 MDHS has been reported in detail elsewhere [1].

### Study variables

#### Outcome variable

The outcome variable for this study was based on a self–reported previous HIV testing status. This was measured by asking the participants this question: ‘Have you ever tested for HIV?’ HIV testing was treated as a binary variable and the responses were coded as *yes* or *no*. The ‘yes’ category included those who had ever tested for HIV at least once in their lifetime prior to this survey and the ‘no’ category included those who had never tested for HIV in their lifetime.

#### Covariates

The potential covariates of HIV testing were selected based on literature review [19–21] and completeness of data within the 2015–2016 MDHS dataset. The selected covariates were the following: age (in four categories: 15–19,20–29, 30–39, 40–54 years), marital status(categorized as never married, married and formerly married), region of residence (northern, central, southern), area of residence (rural vs. urban), education level (none, primary school and secondary school or above), religion(Christians, Muslims and no religion), covered by health insurance (yes/no), age at sexual debut (categorized as: no sex, less than 15 years, 15–24 years,24 and above years) and an asset-based index of wealth(poor, medium and rich) [22]. Some of these study variables were re-coded to suit the purpose of the study while some were used as they are in the original dataset. For instance, religious affiliation was re-coded into Christians, Muslims and no religion. MDHS categorized household wealth index into five groups; poorest, poor, middle, richer, and richest. This study re-coded household wealth index into three groups by combining “poorer” and “poor” for poor and “rich” and “richer” for rich.

### Ethical considerations

The 2015–2016 MDHS data collection procedures were approved by the ICF Macro (Calverton, Maryland), the Malawi’s National Health Sciences Research Committee (NHSRC) and informed consent was obtained from respondents at the start of the individual interviews [1]. Permission to use the data was obtained from Measure DHS, which is a USAID–funded project that assists and fund population and health surveys in countries worldwide. No further ethical approval was necessary since the study was based on anonymous public use data with no identifiable information on survey respondents.

### Statistical analysis

Descriptive statistics (frequencies) were calculated to describe the characteristics of men included in this study and the results were presented as proportion (%). The association between each categorical covariate with the outcome variable (HIV testing) was tested by using Pearson chi-square test (χ2). Logistic regression was then used to establish the magnitude and direction of the associations. All variables that were significant at *P* ≤ 0.25 in bivariate analysis (logistic regression) were entered together into a multiple logistic regression model [23]. Crude and adjusted odds ratios and their 95% confidence intervals (95% CI) were estimated. A complex sample analysis procedure of the Statistical Package for the Social Sciences (SPSS, IBM version 22) was used to account for the multistage sampling used in DHS. All analyses used sampling weights and adjusted for the sampling design (clustering and stratification). A *P-value* < 0.05 was considered statistically significant.

## Results

### Characteristics of the study population and HIV testing uptake

Table 1 presents the weighted profile of men in the analysis sample by their HIV testing category. Of the 7478 men interviewed, 69.9% had tested for HIV. The age of the participants ranged from 15 to 54 years with mean ± SD of 28.9 ± 10.6 years. The younger age group (15–19 years) represented about 24.3%. Majority of the respondents were rural dwellers (81.5%), Christians (86.1%) and were not covered by health insurance (97.2%). Nearly half of the respondents were from central region (44.4%) and 58% were married. More than half (58.5%) of the participants had completed primary level of education and only 6% of the respondents had no formal education. Almost half of the respondents were regarded as rich (45.8%). Bivariate analysis (Table 1) showed that most of the background characteristics of the respondents were significantly associated with HIV testing (*P < 0.001*). However, religion showed a marginal association with HIV testing (*P = 0.046*).

**Table 1:** HIV testing uptake by different background characteristics among men aged 15–54 years who participated in the 2015–2016 Malawi Demographic Health Survey

Characteristics			HIV testing status				
	Total		Never tested		Ever tested		*p-value*
	n	%	n	%	n	%	
Age (years)							< 0.001*
15–19	1818	24.3	1188	52.8	630	12.1	
20–29	2430	32.5	534	23.7	1896	36.3	
30–39	1807	24.2	240	10.7	1567	30.0	
40+	1423	19.0	288	12.8	1135	21.7	
Mean (±SD) age (years)	28.9 ± 10.6						
Residence							< 0.001*
Rural	6096	81.5	1897	84.3	4199	80.3	
Urban	1382	18.5	353	15.7	1029	19.7	
Region							< 0.001*
Northern	966	12.9	236	10.5	730	14.0	
Central	3321	44.4	1049	46.6	2272	43.5	
Southern	3191	42.7	965	42.9	2226	42.6	
Marital Status							< 0.001*
Never married	2866	38.3	1522	67.6	1344	25.7	
married	4347	58.1	676	30.0	3671	70.2	
Formerly married	264	3.5	52	2.3	212	4.1	
Religion							0.046*
Christians	6437	86.1	1917	85.2	4520	86.5	
Muslim	807	10.8	246	10.9	561	10.7	
No religion	233	3.1	87	3.9	146	2.8	
Wealth							< 0.001*
Poor	2572	34.4	800	35.6	1772	33.9	
Medium	1480	19.8	488	21.7	992	19.0	
Rich	3425	45.8	961	42.7	2464	47.1	
Education							< 0.001*
None	448	6.0	142	6.3	306	5.9	
Primary school	4373	58.5	1563	69.5	2810	53.7	
Secondary school or above	2657	35.5	545	24.2	2112	40.4	
Covered by health insurance							< 0.001*
No	7268	97.2	2221	98.7	5047	96.5	
Yes	210	2.8	29	1.3	181	3.5	
Age at first sexual debut (years)							< 0.001*
No sex	1018	13.6	726	32.3	292	5.6	
Less than 15	1077	14.4	401	17.8	676	12.9	
15–24	5075	67.9	1077	47.9	3998	76.5	
24+	308	4.1	46	2.0	262	35.0	

*Significant (*P* < 0.05)

### Determinants of HIV testing among men in Malawi

Correlates of HIV testing among men in Malawi based on unadjusted and adjusted logistic regression are presented in Table 2. The results revealed that age, region of residence, marital status, covered by health insurance, education and age at first sexual are significant predictors of HIV testing among men in Malawi. In particular, men who were in the 30–39 years old age category were three times (AOR = 3.00; 95% CI = 2.35–3.82) more likely to have been tested for HIV than those who were in the age group 15–19 years. Men who had completed primary level of education and secondary or above education were approximately 1.5 times (AOR = 1.49; 95% CI = 1.19–1.88) and 3 times (AOR = 3.02; 95% CI = 2.33–3.91), respectively, more likely to have been tested for HIV as compared to those with no education. Similarly, being married (AOR = 3.03; 95% CI = 2.51–3.65) increased the likelihood of having been tested for HIV than those men who had never married. Men who had first sexual debut at 15–24 years had higher odds of having been tested (AOR = 2.54; 95% CI = 2.11–3.07) than men who never had sex. The uptake of HIV testing was higher among men who were covered by health insurance (AOR = 1.66; 95% CI = 1.05–2.63) compared to men who had no health insurance. Conversely, men who were residing in central region (AOR = 0.64; 95% CI = 0.53–0.78) and southern region (AOR = 0.74; 95% CI = 0.61–0.90) were less likely to have tested for HIV in comparison with men who were residing in the northern part of Malawi.

**Table 2:** Univariate and multivariable logistic regression analyses of independent factors associated with HIV testing among men in Malawi

Characteristics	COR	95% CI	*P-Value*	AOR	95% CI	*P-Value*
Age (Years)						
15–19	1			1		
20–29	6.71	5.85–7.68	< 0.001*	2.57	2.16–3.05	< 0.001*
30–39	12.30	10.41–14.53	< 0.001*	3.00	2.35–3.82	< 0.001*
40+	7.44	6.33–8.75	< 0.001*	2.02	1.58–2.59	< 0.001*
Residence						
Urban	1			1		
Rural	0.76	0.67–0.87	< 0.001*	1.03	0.87–1.23	0.743**
Region						
Northern	1			1		
Central	0.70	0.59–0.83	< 0.001*	0.64	0.53–0.78	< 0.001*
Southern	0.75	0.63–0.88	< 0.001*	0.74	0.61–0.90	0.002*
Marital Status						
Never married	1			1		
Currently married	6.15	5.51–6.87	< 0.001*	3.03	2.51–3.65	< 0.001*
Formerly married	4.63	3.39–6.33	< 0.001*	2.24	1.57–3.18	< 0.001*
Religion						
Christians	1			1		
Muslims	0.97	0.82–1.13	0.674**	1.06	0.88–1.27	0.559**
No religion	0.71	0.54–0.93	0.014*	0.74	0.54–1.01	0.054**
Wealth Index						
Poor	1			1		
Medium	0.92	0.80–1.05	0.215**	0.95	0.81–1.11	0.497**
Rich	1.16	1.04–1.29	0.010*	1.08	0.93–1.25	0.332**
Education						
None	1			1		
Primary school	0.84	0.68–1.03	0.090**	1.49	1.19–1.88	0.001*
Secondary school or above	1.80	1.45–2.25	< 0.001*	3.02	2.33–3.91	< 0.001*
Covered by health insurance						
No	1			1		
Yes	2.76	1.86–4.11	< 0.001*	1.66	1.05–2.63	0.030*
Age at first sexual debut (years)						
No sex	1			1		
Less than 15	4.20	3.50–5.05	< 0.001*	2.12	1.72–2.61	< 0.001*
15–24	9.24	7.94–10.75	< 0.001*	2.54	2.11–3.07	< 0.001*
24+	14.24	10.11–20.05	< 0.001*	2.46	1.69–3.59	< 0.001*

Note Abbreviations: *AOR* adjusted odds ratio, *COR* crude odds ratio. *Significant (*P* < 0.05); **Non-significant (*P* > 0.05)

## Discussion

The study findings demonstrate that over two–third (69.9%) of men in Malawi had been tested for HIV. The results highlight that several socio–demographic, behavioral and health service related factors influence the uptake of HIV testing services among men in Malawi. In particular, participants’ age was an important factor associated with HIV testing in this study. Men who were aged 20 years or older were more likely to have had HIV testing compared to adolescent men (15–19 years). This finding is in accordance with a recent study in Haiti which revealed that older men were more likely to have been tested for HIV than men aged 15 to 19 years [24]. Similarly, a study in South Africa reported that young males were less likely to be tested for HIV than order males [25]. The low uptake of HIV testing among adolescent men observed in this study could be attributed to a number of factors. Adolescent men may have low HIV–related knowledge as well as limited access to health care services including HIV testing services. For instance, a report by UNICEF reveals that only 32% of boys aged 15–19 years in sub–Saharan Africa know how HIV is transmitted and how it can be prevented [26]. Furthermore, men aged 15 to 19 years might have low engagement of sexual behaviours, therefore perceive themselves as having a lower risk of HIV, which in turn contributes to low uptake of HIV testing among this age group. Fear and stigma surrounding HIV and HIV testing in healthcare facilities may also prevent adolescent men from getting tested for HIV in Malawi. Thus, program planners need to develop and design effective programs, policies, and strategies that target adolescent men in Malawi.

The present study demonstrated that having higher educational level was associated with ever being tested for HIV. Educated men might have higher levels of exposure to HIV/AIDS–related information, better knowledge of the advantages of HIV testing as well as ability to make good decision to go for HIV testing than their uneducated counterparts. Other studies conducted elsewhere have also shown that having higher educational status was associated with ever being tested for HIV [17, 19, 27, 28]. These results highlight the importance of providing health education to men with low level or no education. Promotion of education among men is also a potential strategy to increase HIV testing among men in Malawi.

Region of residence was also associated with ever being tested for HIV even after adjusting for other variables. We observed that men residing in central and southern region were less likely to test for HIV than those from northern region. Similar findings have also been reported in other studies conducted in Ethiopia and Mozambique, where regional variations in uptake of HIV testing was observed [27, 29]. Cultural values and lifestyle differences across the regions could partly explain the observed differences in HIV testing. Moreover, regional differences in availability and access to HIV testing services as well as HIV/AIDS–related information could also be the reasons for the observed regional disparities in the uptake of HIV testing. Therefore, there is a need to expand HIV testing services to central and southern region to reach more men.

The results further indicate that HIV testing among men was positively associated with marital status. Men who were married and formerly married were more likely to have been tested for HIV than men who were never married. The possible explanation for this association is that the ministry of health in Malawi encourages male involvement in reproductive health and other care services. As a result, men may have opportunity of being tested for HIV because of frequent interaction with the health care providers when they accompany their partners to the clinic than their unmarried counterparts. Moreover, men are also likely to undergo for HIV testing when they are planning for marriage to know their HIV sero–status than those who have never been married. This result is consistent with the findings of previous studies conducted in Kenya and Mozambique [29, 30].

This study revealed that being covered by health insurance was associated with increased likelihood of HIV testing among Men in Malawi. This finding concurs with the results of a previous study in Haiti which reported that men who had health insurance were likely to have been tested for HIV compared to those who had no health insurance [24]. The most plausible explanation for this relationship is that men with health insurance tend to have access to regular health care and screenings including HIV testing than those who do not have health insurance. Besides, increased use of health care services by men with insurance also increases their chances of being offered the test through frequent encounters with health care providers. Therefore, there is a need to promote interventions such as free community based HIV testing programs to reach men with no health insurance in order to increase HIV testing among men in Malawi.

While the body of existing evidence suggests that key socio–demographic factors such as wealth index, area of residence and religion are associated with HIV testing [28, 31, 32], this study found contrary results. These variables were strongly associated with HIV testing in bivariate analyses; however, in the multivariable model the results modified and were all not significantly associated with HIV testing. These conflicting results could possibly be due to different sample size, study participants and setting as well as the differences in the definition of ‘HIV testing’ adopted by the studies. Further exploration of the role of these factors in uptake of HIV testing among men in Malawi is recommended.

It is imperative that our results be interpreted under a set of limitations. First, the study was limited to only the variables collected in the MDHS. As such, other important variables (such as substance use and sexual orientation) that may affect HIV testing but were not available in MDHS could not be examined. Second, the cross-sectional nature of MDHS limits the capacity to draw any causal inferences. Third, assessment of the outcome variable and some of the covariates was based on self-reported, thereby potentially introducing social desirability bias in participants’ responses. Furthermore, the present study assessed the lifetime occurrence of HIV testing by the participants. It did not focus on recent HIV test seeking behaviour (i.e., last 3 months, last 6 months, last 12 months). It is therefore possible that HIV testing happened before the exposure status. Finally, the outcome measure ‘ever been tested for HIV’, while being useful indicator to assess the overall uptake of HIV testing services, does not assess whether the participants who took HIV test received the results of their test or not. Taking an HIV test without knowing the ‘results of the test’ might not have implication for HIV prevention. Further research is needed to complement the findings of the current study by addressing the above limitations.

Despite these limitations, this study is the first to analyze the correlates of HIV testing among men in Malawi using a nationally representative sample. Given the representativeness of our sample, the current findings are generalizable to the entire country.

## Conclusion

This paper provides nationally representative statistics related to HIV testing among men aged 15–54 years in Malawi. The findings suggest the need for health policy makers as well as other stakeholders to sustain current efforts to target younger unmarried men aged 15–19, men with low level or no education and expand HIV testing services to the central and southern regions of Malawi. Targeting the undiagnosed men living with HIV in a timely manner is a crucial and necessary step not only for achieving the UNAIDS’ 90–90-90 targets but for individuals to benefit from antiretroviral treatment and to sustainably reduce population–level HIV transmission.

## Abbreviations

ART: Antiretroviral Therapy
CDC: Centre for Disease Control
HTS: HIV testing services
MDHS: Malawi demographic health survey
NSO: National statistical office
PMTCT: Prevention of mother-to-child transmission
SSS: Sub–Saharan Africa
UNAIDS: United Nations Programme on HIV/AIDS
UNICEF: United Nations Children’s Fund
USAID: United States Agency for International Development
WHO: World Health Organisation

## References

[1] ICF NSONMa: Malawi Demographic and Health Survey 2015–16. In*.* Zomba, Malawi, and Rockville, Maryland, USA; 2017.

[2] UNAIDS. Contry factsheets: Malawi 2016. Available at: http://www.unaids.org/en/regionscountries/countries/malawi/. Accessed 08 Jan 2019.

[3] Levi J, Raymond A, Pozniak A, Vernazza P, Kohler P, Hill A (2016). Can the UNAIDS 90-90-90 target be achieved? A systematic analysis of national HIV treatment cascades. BMJ Glob Health.

[4] Li J, Gilmour S, Zhang H, Koyanagi A, Shibuya K (2012). The epidemiological impact and cost-effectiveness of HIV testing, antiretroviral treatment and harm reduction programs. Aids.

[5] Fonner VA, Denison J, Kennedy CE, O'Reilly K, Sweat M. Voluntary counseling and testing (VCT) for changing HIV-related risk behavior in developing countries. Cochrane Libr. 2012;9:CD001224. 10.1002/14651858.CD001224.pub4 PMC3931252 22972050

[6] Kagee A, Swartz A, Swartz L (2014). Theorising beyond the individual: adherence to antiretroviral therapy in resource-constrained societies. J Health Psychol.

[7] Organization WH: service delivery approaches to HIV testing and conselling (HTC): a strategic HTC programme framework. 2012.

[8] Woodring JV, Kruszon-Moran D, Oster AM, GM MQ. Did CDC's 2006 revised HIV testing recommendations make a difference? Evaluation of HIV testing in the US household population, 2003–2010, J Acquir Immune Defic Syndr. 2014;67(3):331–40. 10.1097/QAI.0000000000000303 PMC7241860 25153918

[9] HIV/AIDS JUNPo: 90–90-90—an ambitious treatment target to help end the AIDS epidemic. Geneva: UNAIDS; 2014. In*.*; 2017.

[10] Jürgensen M, Tuba M, Fylkesnes K, Blystad A (2012). The burden of knowing: balancing benefits and barriers in HIV testing decisions. A qualitative study from Zambia. BMC Health Serv Res.

[11] De Allegri M, Agier I, Tiendrebeogo J, Louis VR, Yé M, Mueller O, Sarker M (2015). Factors affecting the uptake of HIV testing among men: a mixed-methods study in rural Burkina Faso. PLoS One.

[12] Bwambale FM, Ssali SN, Byaruhanga S, Kalyango JN, Karamagi CA (2008). Voluntary HIV counselling and testing among men in rural western Uganda: implications for HIV prevention. BMC Public Health.

[13] Ministry of Health M: Malawi HIV Testing Services Guidelines. In*.* Lilongwe,Malawi; 2016.

[14] Ministry of Health M: Malawi Population-based HIV Impact Assessment (MPHIA) 2015–16. In*.* Edited by Health Mo. Lilongwe, Malawi; 2017.

[15] Conserve D, Sevilla L, Mbwambo J, King G (2013). Determinants of previous HIV testing and knowledge of partner's HIV status among men attending a voluntary counseling and testing clinic in Dar Es Salaam, Tanzania. Am J Mens Health.

[16] Molla G, Huruy A, Mussie A, Wondowosen T. Factors associated with HIV counseling and testing among males and females in Ethiopia: evidence from Ethiopian demographic and health survey data. Journal of AIDS and Clinical Research. 2015;6(3):429.

[17] Kirakoya-Samadoulougou F, Jean K, Maheu-Giroux M. Uptake of HIV testing in Burkina Faso: an assessment of individual and community-level determinants. BMC Public Health. 2017;17(1):486. 10.1186/s12889-017-4417-2 PMC5441086 28532440

[18] Hensen B, Lewis J, Schaap A, Tembo M, Vera-Hernández M, Mutale W, Weiss H, Hargreaves J, Stringer J, Ayles H (2015). Frequency of HIV-testing and factors associated with multiple lifetime HIV-testing among a rural population of Zambian men. BMC Public Health.

[19] Obermeyer CM, Neuman M, Hardon A, Desclaux A, Wanyenze R, Ky-Zerbo O, Cherutich P, Namakhoma I (2013). Socio-economic determinants of HIV testing and counselling: a comparative study in four African countries. Tropical Med Int Health : TM & IH.

[20] Detsis M, Tsioutis C, Karageorgos SA, Sideroglou T, Hatzakis A, Mylonakis E (2017). Factors associated with HIV testing and HIV treatment adherence: a systematic review. Curr Pharm Des.

[21] Makusha T, Mabaso M, Richter L, Desmond C, Jooste S, Simbayi L (2017). Trends in HIV testing and associated factors among men in South Africa: evidence from 2005, 2008 and 2012 national population-based household surveys. Public Health.

[22] Rutstein SO, Johnson K: The DHS wealth index. DHS comparative reports no. 6. *Calverton: ORC Macro* 2004.

[23] Sun G-W, Shook TL, Kay GL (1996). Inappropriate use of bivariable analysis to screen risk factors for use in multivariable analysis. J Clin Epidemiol.

[24] Conserve DF, Iwelunmor J, Whembolua GL, Sofolahan-Oladeinde Y, Teti M, Surkan PJ (2017). Factors associated with HIV testing among men in Haiti: results from the 2012 demographic and health survey. Am J Mens Health.

[25] Peltzer K, Matseke G (2013). Determinants of HIV testing among young people aged 18 - 24 years in South Africa. Afr Health Sci.

[26] UNAIDS and UNICEF. All in to end adolescent AIDS epidemic, progress report. Available at: http://www.unaids.org/sites/default/files/media_asset/ALLIN2016ProgressReport_en.pdf. Accessed 8 Jan 2019.

[27] Teklehaimanot HD, Teklehaimanot A, Yohannes M, Biratu D (2016). Factors influencing the uptake of voluntary HIV counseling and testing in rural Ethiopia: a cross sectional study. BMC Public Health.

[28] Brima N, Burns F, Fakoya I, Kargbo B, Conteh S, Copas A (2015). Factors associated with HIV prevalence and HIV testing in Sierra Leone: findings from the 2008 demographic health survey. PLoS One.

[29] Agha S (2012). Factors associated with HIV testing and condom use in Mozambique: implications for programs. Reprod Health.

[30] Ziraba AK, Madise NJ, Kimani JK, Oti S, Mgomella G, Matilu M, Ezeh A (2011). Determinants for HIV testing and counselling in Nairobi urban informal settlements. BMC Public Health.

[31] Gage AJ, Ali D (2005). Factors associated with self-reported HIV testing among men in Uganda. AIDS Care.

[32] Leta TH, Sandøy IF, Fylkesnes K (2012). Factors affecting voluntary HIV counselling and testing among men in Ethiopia: a cross-sectional survey. BMC Public Health.

